# Cost-effectiveness analysis of Brolucizumab compared to Aflibercept and Ranibizumab in nAMD with persistent retinal fluid

**DOI:** 10.1177/11206721251388170

**Published:** 2025-10-21

**Authors:** Ricardo García-Serrano Fuertes, Andrés Romero Martínez, Jesús Suarez Pérez, Fernando López Herrero, Margarita Cabanás Jiménez, Antonio Flores Córdoba, José Luis Sánchez Vicente

**Affiliations:** 16885 Hospital Virgen del Rocío, Seville, Spain

**Keywords:** Age-related macular degeneration < retina, developmental abnormalities of vitreous < vitreous / endophthalmitis, choroidal neovascular membranes < UVEA, practice management < socioeconomics and education in medicine/ophthalmology, techniques of retinal examination < retina

## Abstract

**Purpose of the research:**

This study aimed to evaluate the cost-effectiveness of Brolucizumab in patients with exudative age-related macular degeneration (AMD) and persistent retinal fluid unresponsive to previous therapies, within the context of a real-world clinical practice setting in a Spanish referral hospital. Furthermore, the study examined the probabilities of transitioning between therapies.

**Major findings:**

A 6-month treatment projection demonstrated that Brolucizumab was not cost-effective compared to Ranibizumab (incremental cost-effectiveness ratio [ICER]: −12.98) and Aflibercept (ICER: −47.64). Conversely, when assessing only drug and administration visit costs, Brolucizumab appeared cost-effective (ICER of 9.14 versus Aflibercept and 35.01 versus Ranibizumab). The increased burden of follow-up costs, which were €348.96 higher than those for Ranibizumab and €174.48 higher than Aflibercept, likely drove the trend towards non-cost-effectiveness. Additionally, the analysis indicated a 43% probability of transitioning to Faricimab within the studied population.

**Conclusions:**

Brolucizumab was determined to be not cost-effective compared to Ranibizumab and Aflibercept in patients with exudative AMD and persistent retinal fluid, primarily due to a higher number of follow-up visits necessitated by its safety profile. Furthermore, newly observed vitreous opacities and a tendency towards the use of Faricimab were noted.

## Introduction

Age-related macular degeneration (AMD) is a chronic and progressive retinal disease and is the leading cause of blindness in developed countries,^1^ with an incidence rate of 2.4% among individuals over 60 years of age.^2^ The disease diminishes the quality of life for affected individuals and poses a substantial socioeconomic challenge due to increasing life expectancy and various environmental risk factors.^3^ Since the approval of anti-vascular endothelial growth factor (VEGF) pharmacotherapy in 2006, the prevalence of legal blindness and visual disabilities attributed to AMD has significantly decreased, effectively removing neovascular AMD (nAMD) from the list of incurable diseases.^4^

Numerous clinical trials have validated the intravitreal application of anti-VEGF agents for the treatment of nAMD, yielding highly favorable outcomes. These include maintaining best-corrected visual acuity (BCVA) in over 90% of treated patients at 52 weeks, with mean BCVA improvements of 6.5–7.2 letters and significant anatomical enhancements.^5,6^ Patients under treatment necessitate regular monitoring utilizing standardized strategies, such as monthly evaluations or individualized pro re nata (PRN) or treat-and-extend protocols, aimed at aligning treatment burden with patient requirements.^7^ Currently, in Europe, Ranibizumab (Lucentis^®^, Novartis, Switzerland), Aflibercept (Eylea^®^, Bayer, Germany), Brolucizumab (Beovu^®^, Novartis, Switzerland), and Faricimab (Vabysmo^®^, Roche, Switzerland) are the sole drugs sanctioned for the management of nAMD.^7^ The latter two represent more recent entrants, whereas substantial clinical experience exists with the older agents.

Persistent retinal fluid is defined as either subretinal fluid (SRF) or intraretinal fluid (IRF) visualized on optical coherence tomography (OCT) more than three times annually.^8^ Persistent IRF has been independently correlated with poorer long-term visual acuity outcomes and the development of fibrosis.^9^ In contrast, other studies propose that persistent SRF does not adversely impact visual acuity over a two-year follow-up period.^8,10^ Nonetheless, European guidelines advocate for managing nAMD through OCT monitoring, incorporating both qualitative assessments (e.g., intraretinal cystoid spaces, SRF, and retinal pigment epithelium detachment) and quantitative metrics such as central retinal thickness (CRT). Treatment intervals are adjusted or discontinued based on fluid absence on OCT.^3^

In the context of persistent SRF or IRF, Brolucizumab represents an alternative in this setting, having demonstrated non-inferiority to Aflibercept with regard to visual function^11^ and efficacy in switch therapy for patients with persistent retinal fluid following other anti-VEGF therapies.^12^ Additionally, it allows for extended dosing intervals when compared to other anti-VEGF agents.^13^

However, Brolucizumab is associated with an increased risk of adverse events, such as intraocular inflammation, retinal vasculitis, and retinal vascular occlusion, compared to traditional treatments.^14^ It has also been linked to the emergence of vitreous opacities resembling asteroid hyalosis.^15^

Given these factors, the current study sought to evaluate the cost-effectiveness of Brolucizumab in patients with nAMD and persistent retinal fluid from the perspective of routine clinical practice at the Hospital Virgen del Rocío in Seville, Spain. Although Brolucizumab necessitates fewer injections and has exhibited similar or superior efficacy compared to standard treatments, widespread apprehensions regarding its adverse event profile may drive increased follow-up visits, elevating overall treatment costs.

## Methods

A retrospective study was conducted utilizing the clinical records of patients within a real-world clinical practice setting. The incremental cost-effectiveness ratio (ICER), a widely adopted metric in the economic evaluation of healthcare interventions, was employed for data analysis. The study encompassed retrospective data from two years of treatment involving 22 patients (23 eyes) treated within the Ophthalmology Department at Hospital Virgen del Rocío, with treatment initiation occurring between November 2021 and June 2024. All patients were diagnosed with nAMD exhibiting persistent fluid refractory to multiple anti-VEGF therapies and were managed under a “treat-and-extend” protocol.

Patient selection was predicated on a diagnosis of nAMD with persistent IRF or SRF and the commencement of treatment with Brolucizumab following an inadequate response to Ranibizumab or Aflibercept. Some patients subsequently transitioned to Faricimab therapy due to insufficient response to Brolucizumab. The study tracked a total of 24 eyes undergoing Brolucizumab treatment for varying durations.

ICER was computed by dividing the incremental change in costs (Euro-€) by the incremental change in health outcomes, measured as the percentage improvement in BCVA during the treatment period (€/% BCVA improvement). Only patients exhibiting improvement or no deterioration in BCVA were included in the analysis, as AMD is a degenerative condition, negative changes were excluded.

Clinical and economic parameters (quantified in decimal BCVA) were analyzed under real-world conditions, with a BCVA of 0 assigned for visual acuity below “counting fingers”. BCVA measurements were obtained from clinical records before the initiation of each drug and at the conclusion of the respective treatment. Direct costs (€) encompassed the duration of treatment in months for each drug, drug acquisition costs (€) at the hospital, the number of injections administered, and the costs related to drug administration visits and follow-up visits (€) (both with and without OCT evaluations). Data were extracted from patient clinical records without direct intervention regarding patient treatment decisions.

Unit costs and drug prices were sourced from the hospital's Pharmacy and Expenditure divisions, as well as from publicly established prices regulated by the Decree of October 14, 2005, governing public healthcare service fees in the Andalusian Health Service.

**Figure 1.**
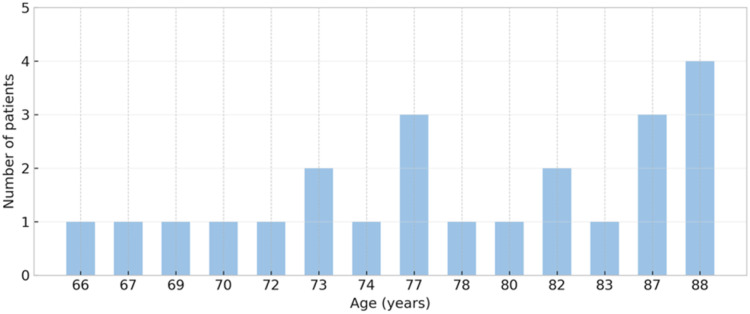
Age distribution of the study sample. The X-axis represents the ages of patients in years (only those with recorded cases are shown). The Y-axis indicates the number of patients at each age.

From the collected data, the mean expenditures on injections, administration visits, and follow-up visits (with and without OCT) were calculated, along with the mean variation in BCVA and its standard deviation, essential for subsequent statistical analysis (Figure 1).

ICER, was calculated for the observed sample. Due to differing treatment durations among patients in real-world clinical practice, this ICER provided a general estimate of the sample's outcomes, offering insights into potential results in alternative scenarios.

To further enhance the relevance of cost-effectiveness comparisons between Brolucizumab and standard treatments (Aflibercept and Ranibizumab), an ICER simulation was conducted based on 6-month treatment regimens for each drug. In this alternative approach, monthly rates of intravitreal injections, follow-up visits, and BCVA variation were calculated for each therapy based on retrospective cohort data. These rates reflected the expected monthly clinical and resource-use changes within the study population. By applying them, a common treatment duration of 6 months was simulated across all therapies, enabling ICER values to be interpreted on a standardized time basis and supporting more concrete and comparable conclusions.

All ICER calculations, for both the observed sample and the 6-month simulation, incorporated total costs and itemized expenses (€), including the number of injections, drug costs (€) combined with administration visit fees (€), and follow-up visit costs (€). This methodology facilitated the identification of the elements most significantly contributing to cost variations.

Lastly, due to the elevated incidence of adverse events associated with Brolucizumab, the study examined the potential influence of these events, including their follow-up and management costs (€), on cost-effectiveness outcomes.

## Results

The medical records of 22 patients and 23 eyes were analyzed over a two-year treatment period for each patient. The sample consisted of 26% females and 74% males, with right eyes representing 52% of cases and left eyes comprising 48%. Of all 23 eyes treated, 43.48% were the best-seeing eyes. The age distribution of the sample is summarized in Figure 1. Most participants were over 70 years of age. (Table 1)

**Table 1.** Breakdown of average expenditures on injections, drug administration visits, and follow-up visits (with and without OCT, alongside the mean variation in BCVA, and its standard deviation (above).

Drug	Avg. injection rate	Avg. revision rate	Avg. av. rate	Months
Aflibercept	3	3	0.05017	6
Brolucizumab	2	3	0.029063	6
Ranibizumab	3	3	0.040987	6

Drug	Avg. cost injections	Avg. injector cost	Avg. revision cost	Avg. av. variation	Sd av. var.
Aflibercept	511.875	206.9497	336.6417	0.050656	0.081692
Brolucizumab	608.6087	156.0261	814.8109	0.03385	0.075792
Ranibizumab	739.0033	263.7633	294.4387	0.02875	0.101061

BCVA: best corrected visual acuity; OCT: optical coherence tomography; AVG: average; AV: visual acuity; Sd: standard deviation; Var: variation. Also depicted are the average semiannual rates of injections, follow-up visits, and BCVA variation over 6 months of treatment, with default approximations made for the decimal values of the average injection and follow-up rates.

### BCVA progression in the sample

**Figure 2.**
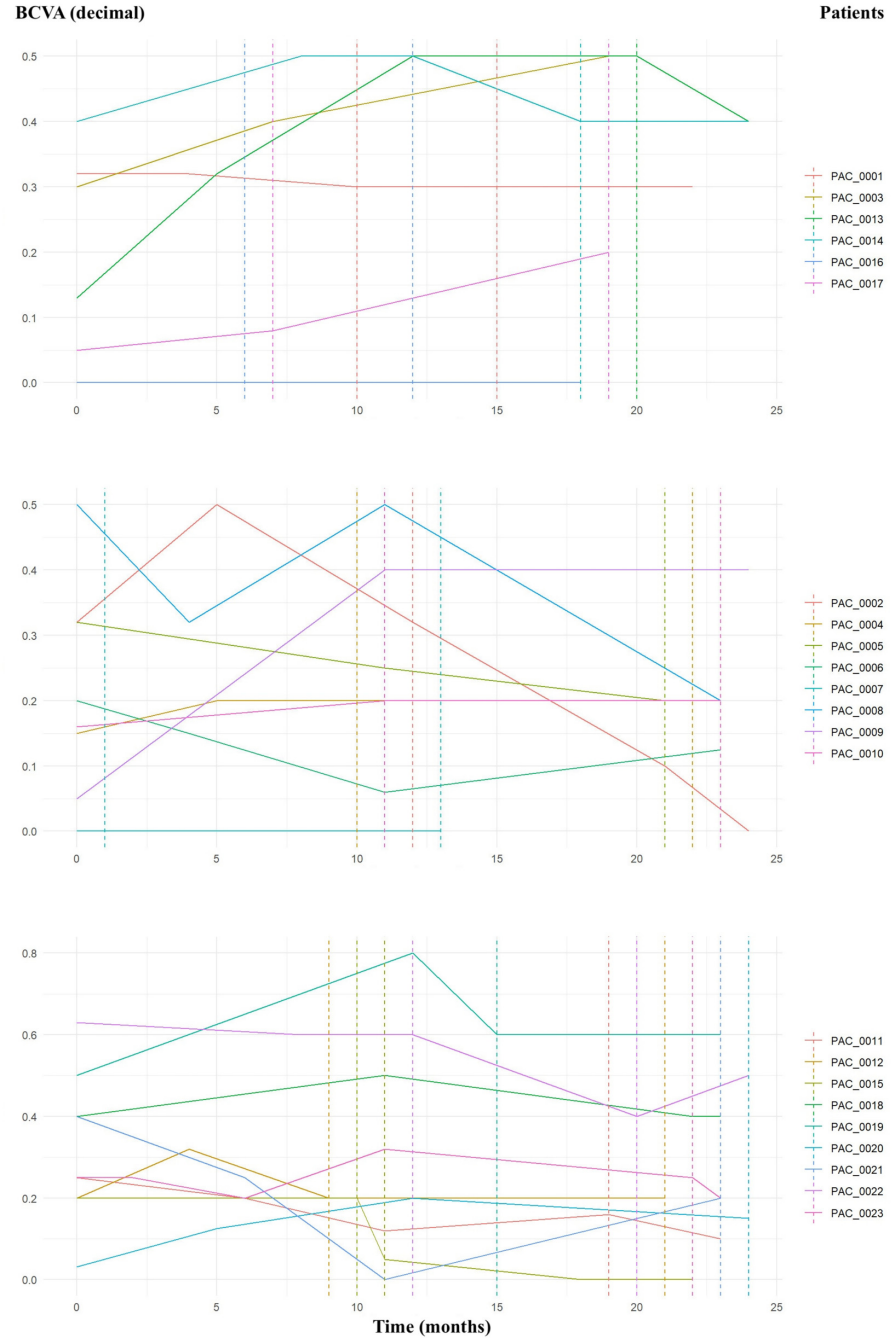
Visual acuity (VA) progression over time for female patients (top). VA progression for 8 male patients over time (middle), and VA progression for the remaining male patients over time (bottom). All visual acuities were measured using a Snellen chart in decimal notation. The start and end of Brolucizumab treatment are marked with dashed lines. Certain dashed lines may not be visible due to overlapping start and end periods among patients. BCVA, best corrected visual acuity.

A thorough examination of visual acuity progression was conducted for each patient throughout the two-year period, highlighting the initiation and conclusion of treatment with Brolucizumab and disaggregating data by sex (Figure 2).

It was observed that only 10 eyes (1 female patient, PAC_0001, and 9 male patients: PAC_0002, PAC_0005, PAC_0006, PAC_0008, PAC_0011, PAC_0015, PAC_0021, PAC_0022, PAC_0023) of the total 23 experienced a decline in BCVA over the two-year treatment period. The majority of the sample showed either improvement or no worsening in BCVA during the follow-up period. During the treatment with Brolucizumab, 10 eyes from 10 individuals (PAC_0014, PAC_0002, PAC_0005, PAC_0008, PAC_0015, PAC_0018, PAC_0019, PAC_0020, PAC_0022, PAC_0023) exhibited worsening BCVA, whereas the remaining eyes either maintained or improved their vision.

### Transition probabilities between drugs

**Figure 3.**
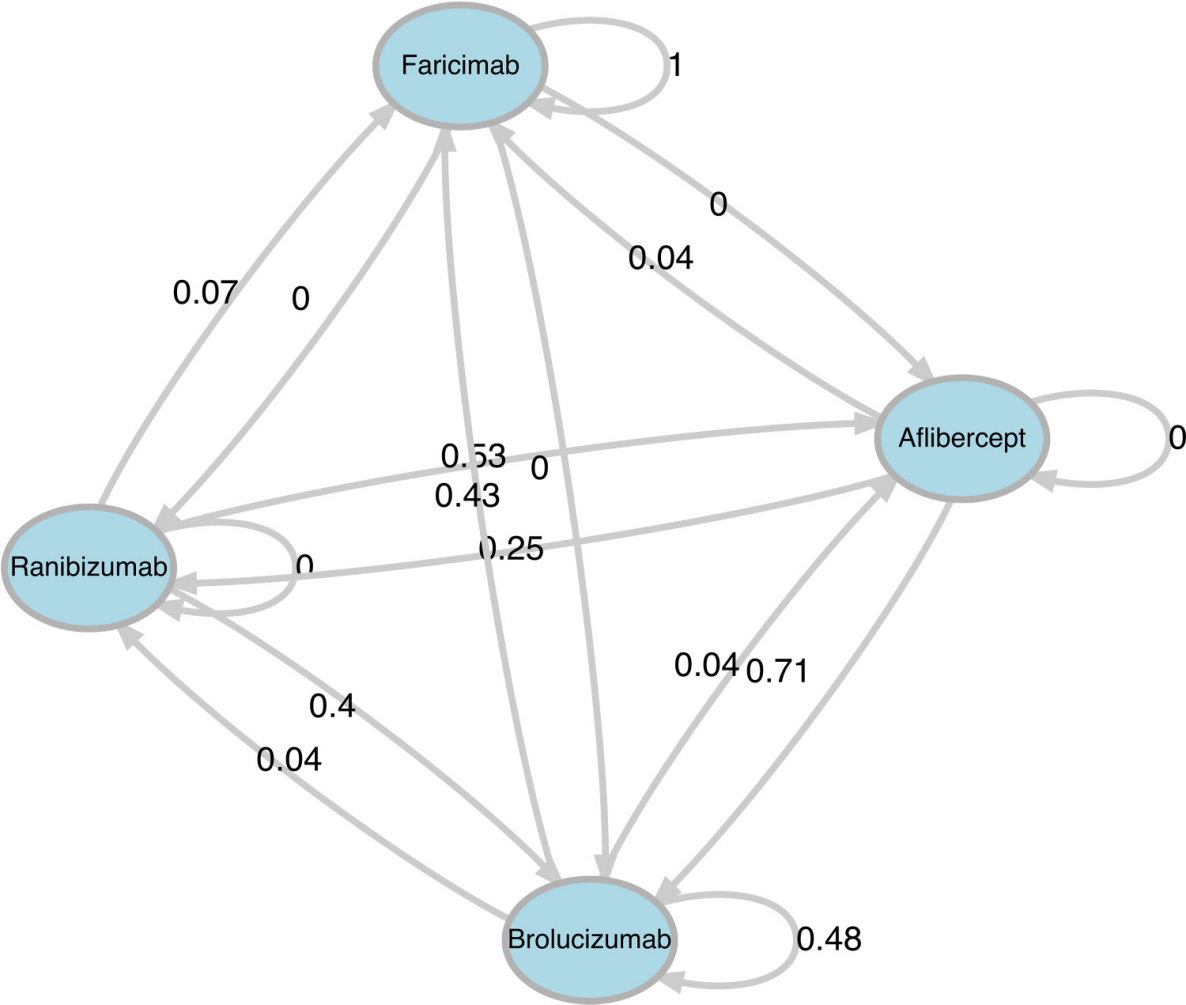
Graphical representation of the transition probabilities in the sample.

By tracking all drug changes in the patients included in the sample, transition probabilities were calculated (Figure 3). These probabilities represent the percentage of patients transitioning between treatments, as follows:

- 40% of patients treated with Ranibizumab transitioned to Brolucizumab.
- 71% of patients treated with Aflibercept transitioned to Brolucizumab.
- Between Aflibercept and Ranibizumab 53% of Ranibizumab-treated patients transitioned to Aflibercept and 25% of Aflibercept-treated patients transitioned to Ranibizumab.
- 48% of Brolucizumab-treated patients continued with the same treatment, while approximately 43% transitioned to Faricimab.

Based on these transition probabilities and observed treatment patterns within our setting, it was inferred that 43% of patients treated with Brolucizumab ultimately transitioned to Faricimab. This finding reflects a prevalent trend in real-world clinical practice. Note that other medications were less likely to transition to Faricimab as patients were selected based on prior treatment with Brolucizumab before switching to other therapies.

In total, 12 patients transitioned to Faricimab, representing 52% of the sample.

### Adverse reactions

Given the increased risk of adverse events related to Brolucizumab treatment, these events were documented in the sample to evaluate their potential effect on the cost-effectiveness analysis.

Within this cohort, no cost increases attributable to adverse effects from Brolucizumab were observed, as none of the aforementioned inflammatory events were reported. However, vitreous opacities not observed prior to treatment were identified in 8 patients (35% of the sample). Despite this occurrence, the presence of vitreous opacities did not affect the frequency of follow-up visits, the necessity for intravitreal treatment, or the overall costs (€).

### Cost-effectiveness results from the sample and 6-month treatment simulation

In the sample analysis, Brolucizumab exhibited lower effectiveness, with 5.98% fewer patients demonstrating improvement or no worsening in BCVA compared to Aflibercept and 10.14% fewer patients compared to Ranibizumab. Moreover, Brolucizumab incurred higher costs, with an average cost increase of €572.53 compared to Aflibercept and €390.75 compared to Ranibizumab. The observed ICER values for Brolucizumab (–95.77 €/% BCVA improvement, relative to Aflibercept and −38.52 €/% BCVA improvement, relative to Ranibizumab) indicated that Brolucizumab was a dominated strategy compared to the other drugs analyzed in this sample.

In the 6-month treatment simulation, discrepancies in costs and effectiveness were less pronounced: 3.07% more patients showed improvement or no worsening in BCVA with Aflibercept, and 7.27% more patients with Ranibizumab, compared to Brolucizumab. Even when treatment durations were equivalent, the average cost of Brolucizumab remained higher—€94.38 more than Ranibizumab and €146.40 more than Aflibercept. Brolucizumab persisted a dominated strategy, as the ICER values remained negative (–12.98 €/% BCVA improvement, compared to Ranibizumab and −47.64 €/% BCVA improvement, compared to Aflibercept), indicating that for each additional patient exhibiting improvement or no deterioration in BCVA, Brolucizumab was associated with cost increases of €12.98 versus Ranibizumab and €47.64 versus Aflibercept in the 6-month projection.

### Analysis with disaggregated costs

#### Breakdown of the drug and administration visit by cost

When only considering drug costs and administration visit expenses, Brolucizumab remained a dominated strategy compared to Aflibercept, offering less improvement or no worsening in BCVA for each euro spent (ICER of −7.43 €/% BCVA improvement, in favor of Aflibercept). However, Brolucizumab was deemed more cost-effective than Ranibizumab, with an ICER of 17.71 €/% BCVA improvement.

Effectiveness continued to favor Aflibercept and Ranibizumab, with 5.98% and 10.14% more patients, respectively, demonstrating improvement or no worsening in BCVA compared to Brolucizumab. Brolucizumab was, on average, €44.41 more expensive than Aflibercept but €179.63 less expensive than Ranibizumab within the sample.

In the 6-month treatment projection, Brolucizumab exhibited lower average costs than both Aflibercept (€28.08 less) and Ranibizumab (€254.58 less). Despite its higher unit cost, the reduced frequency of injections associated with Brolucizumab (averaging 3 injections over 10 months) resulted in overall decreased costs. Based on the data acquired, a 6-month treatment projection that considered only drug injection costs, excluding follow-up visit expenses, determined Brolucizumab to be cost-effective compared to Aflibercept, supported by a positive ICER (9.14 €/% BCVA improvement, in favor of Brolucizumab). Although Aflibercept demonstrated greater effectiveness, with 3.07% more patients showing improvement or no worsening in BCVA compared to Brolucizumab, Brolucizumab still emerged as cost-effective when specific costs were analyzed.

Ranibizumab continued to exhibit lower cost-effectiveness, primarily due to its elevated average cost, with an ICER of 35.01 €/% BCVA improvement, in favor of Brolucizumab. Nonetheless, Ranibizumab maintained superior effectiveness, with 7.27% more patients displaying improvement or no deterioration in BCVA compared to Brolucizumab.

**Figure 4.**
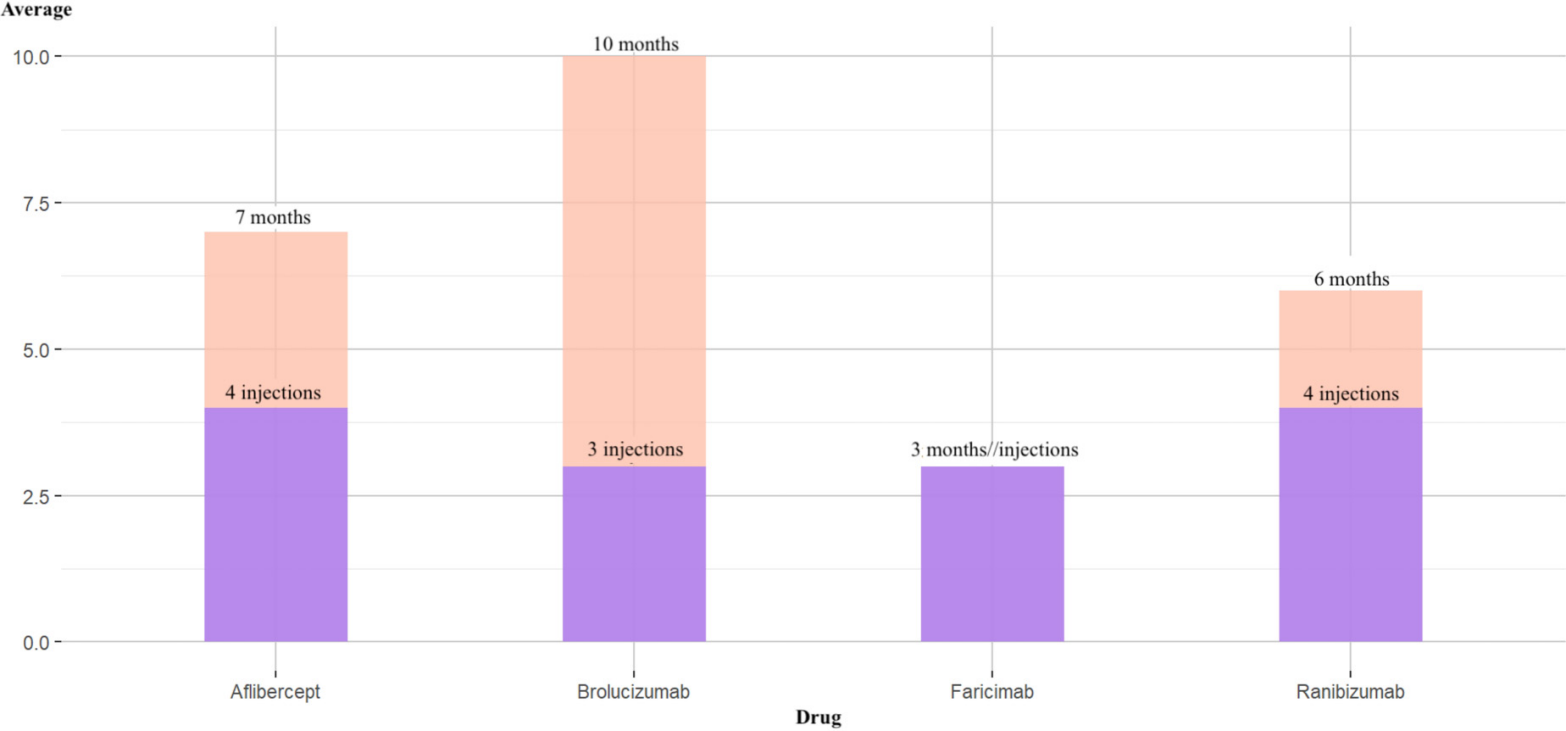
Average number of injections versus average number of months on treatment in the sample.

It was noted that the average treatment duration and the average number of injections were significantly higher for Brolucizumab compared to the other therapies (Figure 4); therefore, treatment with Brolucizumab resulted in a fewer number of injections in our sample, providing support for a reduction in costs associated with each injection or administration event.

#### Breakdown of costs for follow-up visits

When exclusively evaluating the costs of follow-up visits, Brolucizumab exhibited a significant cost increment, averaging €570.38 more than Ranibizumab and €528.12 more than Aflibercept. Effectiveness remained superior for Ranibizumab and Aflibercept, with 10.14% and 5.98% more patients, respectively, indicating improvement or no worsening in BCVA compared to Brolucizumab. This led to highly negative ICER values of −56.22 €/% BCVA improvement, and −88.34 €/% BCVA improvement, when compared with Ranibizumab and Aflibercept, respectively, illustrating that Brolucizumab was a dominated strategy, revealing a more pronounced disparity than observed in the total cost analysis.

A similar yet less extreme pattern emerged in the 6-month treatment projection. The ICER remained negative, calculated at −47.99 €/% BCVA improvement, in favor of Ranibizumab and −56.78 €/% BCVA improvement, in favor of Aflibercept. Costs associated with follow-up visits continued to be elevated, leading to average costs for Brolucizumab that were €348.96 more than Ranibizumab and €174.48 more than Aflibercept. Effectiveness continued to favor Aflibercept with 3.07% more patients demonstrating improvement or no worsening in BCVA, whereas Ranibizumab had 7.27% more patients exhibiting similar outcomes.

**Figure 5.**
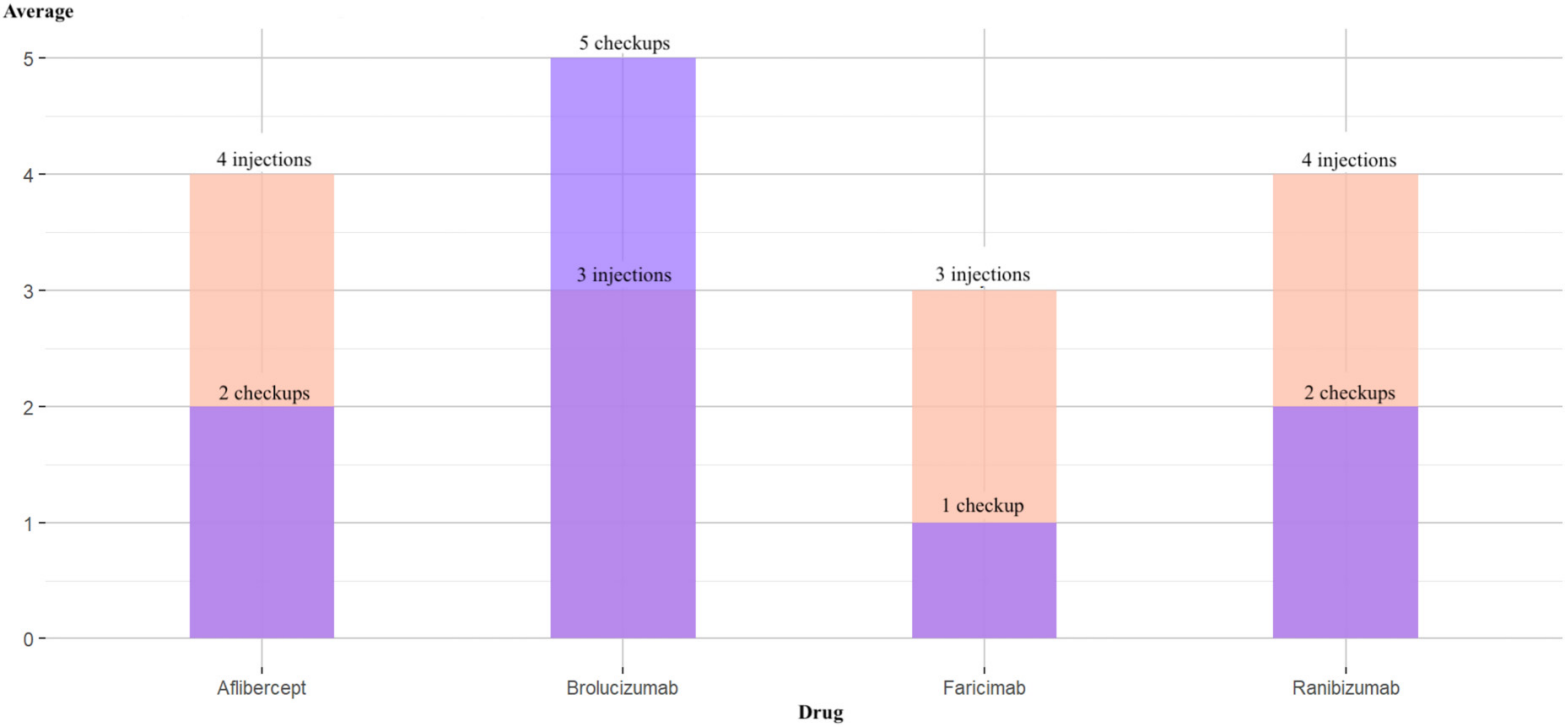
Average number of follow-up visits versus average number of injections in the sample.

It was observed that Brolucizumab was the only drug in which the average number of follow-up visits exceeded the average number of injections compared to the other administered drugs (Figure 5). The sampled cohort indicated that patients treated with Brolucizumab underwent more follow-up visits per injection than those receiving other treatments.

Despite these findings, numerous studies support the effectiveness of Brolucizumab compared to Aflibercept and Ranibizumab for nAMD, inapplicable to both treatment-naïve individuals and in switch scenarios for those with persistent fluid.^7,12,16–18^ Additionally, prior research on cost-effectiveness comparing Brolucizumab to Aflibercept and Ranibizumab among treatment-naïve patients has been published, including in healthcare systems analogous to the Spanish model, which upheld the cost-effectiveness of Brolucizumab.^1,19^

To date, no cost-effectiveness analyses have specifically addressed Brolucizumab usage in treatment-switch scenarios for patients experiencing nAMD with persistent IRF or SRF. This study, therefore, provides valuable insights into this particular patient cohort, which, at least in our context, represents the most frequently considered group for Brolucizumab treatment.

Furthermore, vitreous opacities were noted in 35% of patients, as previously described by Lee KH et al.^15^ Additionally, estimated probabilities of drug-switching based on our sample reflected a 43% likelihood of transitioning to Faricimab, indicating an emerging trend in treatment strategies warranting further exploration in future investigations.

Several limitations must be acknowledged regarding this study. First, the sample size was small and study design was retrospective in nature. Second, all the patients presented with nAMD and persistent fluid, where prior refractoriness to treatments may restrict the effectiveness of the drugs under examination. Third, most patients were treated for 6 months to 1 year with therapies other than Brolucizumab. This suggests that the recorded BCVA outcomes could have been better 6 months or even a year earlier (with Aflibercept or Ranibizumab) than after months of disease progression. This temporal aspect could undermine the effectiveness results obtained with Brolucizumab, often classified as a second-line therapy. Fourth, costs associated with Brolucizumab may have been overestimated in the sample, as an inclusion criterion necessitated prior usage of the drug. This means that all patients received treatment with Brolucizumab, contributing to an increase in the average expenditure attributed to this therapy. Fifth, due to the limited time horizon, the analysis may not fully capture the long-term benefits and costs of treatment. Finally, potential social costs resulting from treatment were not considered, nor were adjustments for quality of life factored into the analysis.

## Discussion

This cost-effectiveness analysis aimed to evaluate whether treatment with Brolucizumab for patients with nAMD and persistent retinal fluid unresponsive to other therapies is cost-effective in routine clinical practice at a regional referral hospital in Spain. Over a two-year follow-up period, which encompassed variable treatment durations, along with a simulated 6-month projection for each drug, Brolucizumab was generally found not to be cost-effective compared to Ranibizumab and Aflibercept.

Although the treatment durations varied among the sampled drugs, the outcomes were consistent with those from the simulated 6-month treatment scenario. The data revealed that the increased costs associated with follow-up visits were instrumental in Brolucizumab's lower cost-effectiveness, despite the drug permitting extended intervals between injections relative to Aflibercept and Ranibizumab. Both the sample data and the 6-month projection indicated additional costs resulting from an increased number of follow-up visits within the Brolucizumab group, contributing to a significantly negative ICER (€/% BCVA improvement). This implies that Brolucizumab incurred higher costs per improvement or lack of deterioration in BCVA when compared to Ranibizumab and Aflibercept.

This investigation appears to be the first documented study concluding that Brolucizumab is not cost-effective when compared to Ranibizumab and Aflibercept in what represents its most common use in routine clinical practice in our region: treating patients with nAMD and persistent retinal fluid.

Additionally, the study identified previously undocumented vitreous opacities in 35% of the patient population. A notable growing trend towards the utilization of Faricimab, a relatively novel drug in the European market, was also observed. This highlights the potential necessity for further research into the effectiveness and costs associated with Faricimab, as well as with Ranibizumab and Aflibercept biosimilars, to thoroughly evaluate their suitability for integration into our healthcare framework.
